# Chatbot-Led Support Combined With Counselor-Led Support on Smoking Cessation in China: Protocol for a Pilot Randomized Controlled Trial

**DOI:** 10.2196/58636

**Published:** 2024-09-23

**Authors:** Xue Weng, Hua Yin, Kefeng Liu, Chuyu Song, Jiali Xie, Ningyuan Guo, Man Ping Wang

**Affiliations:** 1 Institute of Advanced Studies in Humanities and Social Sciences Beijing Normal University Zhuhai China; 2 Office of Clinical Research Administration Zhuhai People’s Hospital (Zhuhai Hospital Affiliated With Jinan University) Zhuhai China; 3 Pulmonary and Critical Care Medicine Guangdong Provincial Hospital of Chinese Medicine Zhuhai China; 4 School of Sociology Beijing Normal University Beijing China; 5 School of Nursing Shanghai Jiao Tong University Shanghai China; 6 LKS Faculty of Medicine, School of Nursing The University of Hong Kong Hong Kong SAR China

**Keywords:** chatbot, smoking cessation, mHealth, mobile phone, campus, China

## Abstract

**Background:**

China has a large population of smokers, with half of them dependent on tobacco and in need of cessation assistance, indicating the need for mobile health (mHealth) to provide cessation support.

**Objective:**

The study aims to assess the feasibility and preliminary effectiveness of combining chatbot-led support with counselor-led support for smoking cessation among community smokers in China.

**Methods:**

This is a 2-arm, parallel, assessor-blinded, pilot randomized controlled trial nested in a smoke-free campus campaign in Zhuhai, China. All participants will receive brief face-to-face cessation advice and group cessation support led by a chatbot embedded in WeChat. In addition, participants in the intervention group will receive personalized WeChat-based counseling from trained counselors. Follow-up will occur at 1, 3, and 6 months after treatment initiation. The primary smoking outcome is bioverified abstinence (exhaled carbon monoxide <4 parts per million or salivary cotinine <30 ng/mL) at 6 months. Secondary outcomes include self-reported 7-day point prevalence of abstinence, smoking reduction rate, and quit attempts. Feasibility outcomes include eligibility rate, consent rate, intervention engagement, and retention rate. An intention-to-treat approach and regression models will be used for primary analyses.

**Results:**

Participant recruitment began in March 2023, and the intervention began in April 2023. The data collection was completed in June 2024. The results of the study will be published in peer-reviewed journals and presented at international conferences.

**Conclusions:**

This study will provide novel insights into the feasibility and preliminary effectiveness of a chatbot-led intervention for smoking cessation in China. The findings of this study will inform the development and optimization of mHealth interventions for smoking cessation in China and other low- and middle-income countries.

**Trial Registration:**

ClinicalTrials.gov NCT05777005; https://clinicaltrials.gov/study/NCT05777005

**International Registered Report Identifier (IRRID):**

DERR1-10.2196/58636

## Introduction

China has the world’s largest population of tobacco users, with more than 300 million current smokers [1]. Approximately half of these smokers are dependent on tobacco and require cessation assistance [2]. Traditional smoking cessation programs, including pharmacotherapy and behavioral counseling [3], have shown efficacy but face challenges such as limited accessibility and high costs in China [4,5], highlighting the need for innovative and scalable interventions.

Mobile health (mHealth) interventions have proven effective in increasing smoking abstinence [6] by enhancing perceived behavioral and psychosocial support for quitting [7]. However, most current mHealth studies rely on fixed-schedule text messages [8-10], which may limit personalization and engagement [11]. Mobile instant messaging (IM) apps (eg, WeChat or WhatsApp) offer more tailored and interactive behavioral and psychosocial support between smokers and smoking cessation counselors. Our previous trials in China found that personalized cessation support via IM, combined with a brief intervention, significantly increased both smoking cessation service utilization and smoking cessation rates [12]. Our pilot trial indicated the feasibility and preliminary efficacy of integrating personalized counselor support via IM with pharmacotherapy [13]. Other trials in China have corroborated these findings, showing the efficacy of IM-based cessation support in increasing abstinence rates [14-16].

Nonetheless, high attrition rates remain a challenge in mHealth interventions. A meta-analysis found a 40%-50% dropout rate in mHealth interventions [17], and our studies observed suboptimal adherence to real-time counselor support, with only about 17% of participants engaging in real-time communication with counselors [12,18]. This limited engagement may be attributed to counselors being available only during working hours, thereby reducing the timeliness and convenience of the support.

Chatbots, automated conversational agents that leverage artificial intelligence and natural language processing to simulate human-like conversations [19], offer a novel opportunity for delivering personalized and interactive cessation support at scale. Unlike human counselor support, chatbots are available 24/7, providing personalized and instant feedback and facilitating psychosocial support [20]. Prior research on chatbot interventions for smoking cessation has shown promise, particularly in enhancing user engagement and adherence to cessation programs [21]. However, our previous trial, which integrated chatbot support with pharmacotherapy and real-time counselor support, did not significantly improve abstinence rates compared with general health messages [22]. The evidence of the effectiveness of chatbots for smoking cessation is not conclusive, with generally small effect sizes [20]. More research is needed on chatbot interventions to determine their efficacy in smoking cessation, particularly in combination with other support modalities in real-world settings.

This study aims to evaluate the integration of chatbot-led support with counselor-led support in promoting smoking cessation among Chinese smokers. The specific objectives of the study are to (1) assess the feasibility of a chatbot-led support intervention for smokers in China, and (2) compare the preliminary efficacy of chatbot-led support combined with counselor-led support versus chatbot-led support alone.

## Methods

### Study Design

**Figure 1:**
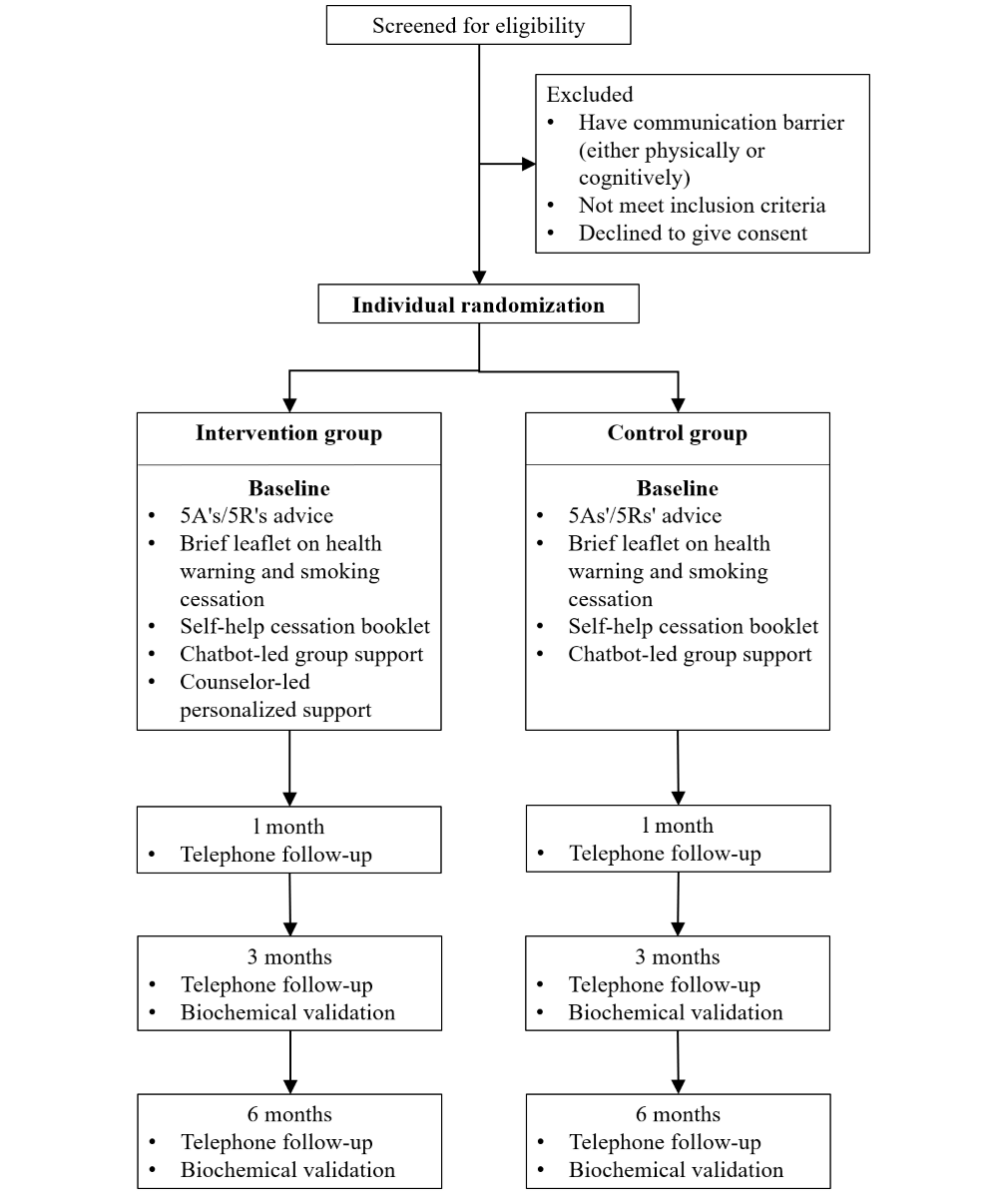
CONSORT (Consolidated Standards of Reporting Trials) flow diagram.

This is a 2-arm, assessor-blinded, pilot randomized controlled trial nested in a smoke-free campus campaign in Zhuhai, China. The campaign is jointly organized by the Youth League and the Staff Union of the Beijing Normal University, Zhuhai, and aims to promote smoking cessation and health education among students and staff [23]. The campaign includes a series of activities, including a “Health for the Future” Smoke-free Promotion Competition that allows students to design publicity materials, a “Smoke-free Ambassador” Competition in which smoke-free volunteers can receive training and recognition, and a “Smoke-free Campus” Smoking Cessation Contest that motivates smokers to quit. The design adheres to the CONSORT (Consolidated Standards of Reporting Trials) statement recommendations (Figure 1) [24].

### Lay Counselor Recruitment and Eligibility

Similar to our previous trials [25-27], university students will be recruited and trained as lay counselors to provide brief cessation advice to community smokers. Recruitment will be advertised through the website and WeChat official account of the university’s volunteer organizations. Eligibility criteria for the lay counselors are as follows: (1) current university students aged 18 years and older; (2) the time and commitment to attend the training sessions and pass the posttraining assessments; and (3) the ability to provide informed consent.

### Participant Recruitment and Eligibility

Web-based promotional strategies will be used to recruit participants through the social media websites of student associations, posters and flyers, and referrals. On-site recruitment activities will be conducted in high-traffic areas on campuses, such as the campus canteen and dormitories. Trained counselors will proactively approach smokers using the “foot-in-the-door” approach [28]. This approach has been extensively used in our previous community trials [25-27] and involves engaging smokers, explaining the study, and inviting them to join the study. The counselors will provide detailed information about the study and the benefits of quitting and the incentives offered. Participants will receive a cash incentive of RMB 100 (approximately US $14) for passing the biochemical validation at 3 and 6 months.

Participant eligibility criteria are intended to be inclusive to maximize generalizability and reach. Eligibility criteria include age of 18 years and older; daily smokers, defined as those smoking ≥1 cigarette per day in the past 3 months; exhaling carbon monoxide ≥4 ppm, indicating active smoking; having a smartphone with WeChat installed; and being motivated to quit smoking or reduce smoking, as assessed using the Transtheoretical Model. Smokers with physical or cognitive communication difficulties or those in other smoking cessation programs will be excluded. All eligible smokers willing to participate will sign a written informed consent form.

Smokers will be informed that the intervention involves a baseline assessment of their exhaled carbon monoxide level, brief questions on past smoking behaviors (baseline questionnaire), and further telephone interviews (follow‐up questionnaires at 1, 2, 3, and 6 months).

### Randomization and Blinding

Participants will be individually randomized in a 1:1 ratio to the intervention or control group. The allocation of participants to the intervention and control groups will be conducted using a computer-generated randomization list. To ensure balance, a randomized blocking schema with block sizes of 2, 4, or 6 will be implemented. Blinding is not possible for the interventionists and participants due to the nature of the intervention. Outcome assessors and statistical analysts will remain masked until the predefined analyses are completed.

### Sample Size

There is no fixed rule for calculating sample size for a pilot study [29]. Prior research on the estimation of sample sizes for pilot studies indicates that a sample of 50 participants per group should be sufficient to achieve 80% power to detect differences between groups [30] on the assumption of a 5% significance level and a 10% attrition rate. Therefore, a total of 150 participants will be recruited for this study.

### Interventionist Training and Supervision

All lay counselors will be required to attend a workshop before participant recruitment. The training covers three key domains: (1) an overview of the intervention content including the 5A/5R advice, chat-led support and counselor-led support, with a demonstration of recruitment strategies such as the foot-in-the-door approach, and the use of exhaled carbon monoxide tests; (2) detailed knowledge of the harms of smoking and the benefits of quitting; and (3) smoking cessation methods and counseling techniques. A pre- and posttest survey will evaluate changes in counselors’ knowledge, attitudes, and practices related to smoking cessation, with only those passing the posttraining assessment qualifying for participant recruitment.

Experienced research staff will provide supervision and assistance at each recruitment session. Counselor performance will be monitored through regular supervision sessions, participant feedback, and periodic reviews of brief counseling by experienced supervisors. Eligible smokers who decline to participate will be asked to provide their reasons for refusal. Information on the number of approached smokers will be gathered, and smokers’ reasons for declining will be recorded verbatim by the smoking cessation counselors. Weekly group supervision will be conducted to review all active cases.

### Interventions

#### Brief Cessation Advice

All participants will receive 5A/5R cessation advice delivered by well-trained cessation counselors on site. The 5A/5R model consists of 5 steps: ask, advise, assess, assist, and arrange for those who are willing to quit in 30 days and relevance, risks, rewards, roadblocks, and repetition for those who are unwilling to quit in 30 days. The participants will also receive a leaflet and a booklet that contain information and tips to help them quit smoking. The leaflet highlights the absolute risk of death due to smoking, diseases caused by active and passive smoking, explicit pictorial health warnings, the benefits of quitting, and motivational messages. The booklet was designed by the Chinese Center for Disease Control and Prevention and includes topics such as benefits and methods of quitting, relapse prevention strategies, and common myths and facts about smoking.

#### Chatbot-Led Group Support

All participants will join a WeChat group led by a chatbot called the “BNU Cessation Assistant.” The chatbot will greet each participant by name and introduce its functions when the participants join the group. The chatbot will engage the participants in interactive conversations by presenting them with a choice of fixed conversations that will cover topics such as the harms of smoking, benefits and methods of quitting, withdrawal symptoms, cessation medications, and quitting self-efficacy. The chatbot will also provide open-ended conversations using natural language processing techniques and will be accessible at any time. The chatbot will deliver announcements and reminders and prompt the participants to share their stories, difficulties, and achievements with other group members. The participants can also communicate with each other for mutual support and encouragement.

The participants can participate in a smoke-free punch-in activity in the group every day to show their commitment and determination to quit smoking. The punch-in activity asks participants to report their smoking status on that day. If a participant reports being smoke-free, he or she will receive encouragement from the chatbot along with the participant’s number of smoke-free days and ranking among other group members. The aim of the punch-in activity is to motivate the participants to quit smoking by increasing their self-efficacy and providing social support.

#### Counselor-Led Personalized Support

Participants in the intervention group will receive 3-month, counselor-led, personalized support through the WeChat platform. This support consists of 2 main components: individualized counseling and prescheduled messages. First, individualized counseling will be provided through one-on-one sessions with trained counselors proficient in behavior change techniques [31]. The counseling is designed to be both proactive and responsive, with counselors initiating regular check-ins and providing tailored advice via text messages. Participants can also initiate contact with counselors as needed. The counselors will respond promptly to any cessation-related queries within working hours (weekdays from 9 AM to 6 PM). Second, participants will receive 24 prescheduled messages over the 3-month period that match their readiness to quit. These messages are delivered on a tapering schedule: 3 messages per week in the first month, 2 messages per week in the second month, and 1 message per week in the third month. The content of these messages includes education on the harms of smoking, the benefits and methods of quitting, techniques for managing cravings, and information on available cessation services. The messages are designed to initiate conversation, and participants are invited to respond.

### Procedures

**Figure 2:**
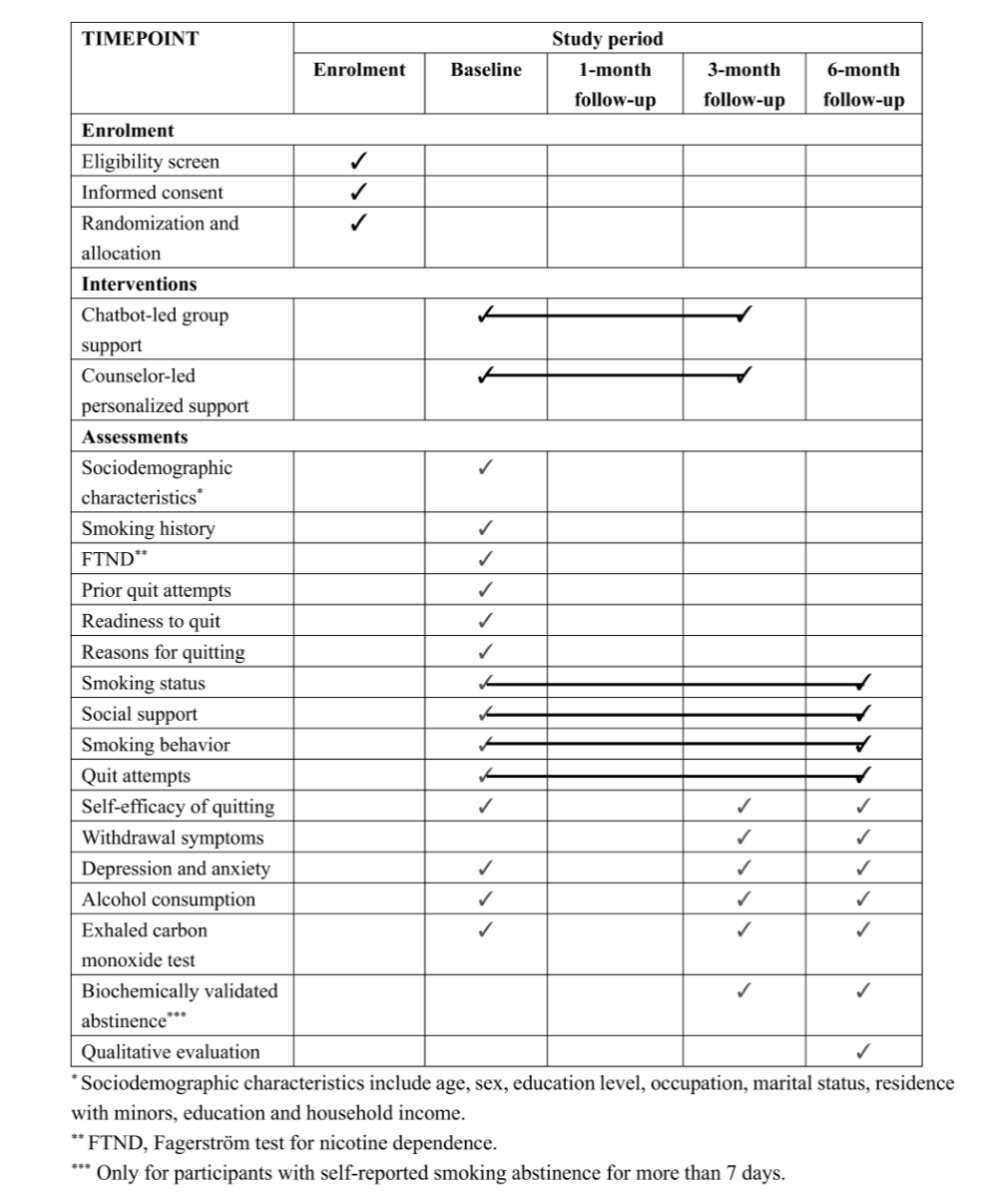
Schedule of enrollment, baseline, and follow-up assessments.

The participants will be assessed at baseline and 1, 3, and 6 months after treatment initiation (Figure 2). The baseline questionnaire will measure the participants’ smoking behavior (eg, daily cigarette consumption, age when starting smoking, time of first cigarette upon waking up in the morning, and quit attempts), readiness to quit, reasons for quitting, social support, perceived self-efficacy of quitting (importance, difficulties and confidence), depression and anxiety, alcohol consumption, and sociodemographic characteristics. The participants will be informed that they may withdraw from the study at any time without giving a reason. The participants will be followed up at 1, 3, and 6 months by trained smoking cessation counselors with a maximum of 7 telephone calls at different times. All data will be stored in a secure, password-protected database. Only authorized research staff will have access to the data. All personal identifiers will be removed from the data set before analysis to ensure confidentiality. Participants who self-report abstinence for more than 7 days at 3 and 6 months will be invited for biochemical validation. Exhaled carbon monoxide samples will be collected by research staff with a piCO Smokerlyzer (Bedfont Scientific Ltd), and saliva cotinine samples will be measured using a test strip (Shanghai Yuduo Biotechnology Co, Ltd). To increase participation, participants will receive a cash incentive of RMB 100 (≈US $14) for passing the biochemical validation at 3 and 6 months.

### Outcomes

#### Smoking Outcomes

The primary smoking cessation outcome is biochemically validated 7-day point prevalence abstinence at 6 months, defined as not smoking (even a puff) in the previous 7 days, confirmed using exhaled carbon monoxide concentrations of <4 ppm or salivary cotinine concentrations of <30 ng/mL. Secondary outcomes include biochemically validated abstinence at 3 months, 7-day point prevalence abstinence, and smoking reduction, defined as at least a 50% reduction in baseline cigarette consumption.

#### Feasibility Outcomes

Feasibility will include the following measures: eligibility rate (the percentage of eligible smokers out of the total number of smokers screened), consent rate (the percentage of eligible smokers who agree to participate out of the total number of eligible smokers), intervention engagement (the percentage of participants who actively engage with the WeChat-based chatbot group, join the smoke-free punch-in activity, read text messages, and chat with counselors), and retention rate (the percentage of participants who remain in the study out of the total number of participants randomized). In addition, participants who engage with the intervention will be asked to rate its effectiveness in increasing their motivation to quit smoking on a scale from 0 (not effective at all) to 10 (very effective).

### Statistical Analyses

Descriptive statistics will be used to summarize the baseline characteristics and outcomes of the participants. Differences between the intervention and control groups will be assessed using chi-square tests for categorical variables and independent *t* tests for continuous variables. The intervention effect on primary and secondary cessation-related outcomes will be analyzed using regression models with and with no adjustment for imbalanced baseline characteristics. Sensitivity to missing data will be examined using multiple imputation by chained equations assuming that the data will be missing at random [32]. The intervention effect by subgroups will be assessed, including age group, sex, education level, household income, previous quit attempts, cigarette dependence, and intention to quit.

Qualitative interview data related to the process and outcomes of the intervention will be analyzed using the thematic framework [33]. The framework will be based on topics specified in the interview guide and emerging themes identified through a process of familiarization with the transcripts. All interviews will be audio-recorded and transcribed verbatim. Up to 10 smoking cessation counselors and 20 participants will be included, depending on data saturation.

### Ethical Considerations

This study was approved by the institutional review board of the University of Beijing Normal University (SSDPP-HSC-20230012). All participants gave their informed consent before taking part in the study. To ensure privacy, all data were anonymized, with unique identifiers removed during processing and only aggregate data used in analysis. Safeguards, including encrypted storage and restricted access, were in place to protect confidentiality. Participants received a cash incentive of RMB 100 (≈US $14) after passing biochemical validation at 3 and 6 months as appreciation for their time and contributions to the research.

## Results

Data collection for the study started in March 2023 and will be completed in June 2024. Findings are expected to be published in high-impact peer-reviewed journals and presented at local, national, and international conferences after November 2024.

## Discussion

This pilot randomized controlled trial aims to examine the feasibility and preliminary effectiveness of a combined chatbot-led smoking cessation intervention in community settings in China. We hypothesize that integrating chatbot support with personalized counselor-led support will improve smoking cessation outcomes. If proven effective, this intervention could offer a scalable, cost-effective, and accessible approach to smoking cessation, addressing the high prevalence of smoking in China. The anticipated findings will contribute to the growing body of evidence on the use of Artificial intelligence–powered chatbots for health promotions and inform the development of a full-scale trial and provide insights for developing more engaging and effective mHealth interventions. The results will also offer valuable strategies for optimizing the use of chatbot interventions, in combination with other support modalities, to enhance smoking cessation outcomes.

This trial is important and innovative for several reasons. First, this trial is being conducted in China, a country with a large number of smokers [1] and regional disparities in access to smoking cessation services [4,14]. Furthermore, most available smoking cessation services are hospital-based [5], which may pose barriers of accessibility and convenience for community smokers. We aim to develop a low-cost, sustainable, and scalable mHealth intervention that can extend cessation services so that community smokers can access them more easily and conveniently. Second, the intervention design built on our previous mHealth trials in China [12,18,27], which showed the effectiveness of real-time counselor support on smoking cessation. By integrating chatbot support, which can provide instant, personalized, and interactive feedback to smokers, we aim to increase mHealth intervention engagement. Third, the study uses a proactive recruitment method by applying the “foot-in-the-door” approach [28], which can recruit a more representative sample of community smokers.

This study has several limitations that should be acknowledged. First, the sample size in this study can assess only the preliminary effectiveness of the intervention. Thus, all results should be treated as preliminary, and conclusive results await a fully powered trial. Second, the absence of a control group without a chatbot-led intervention limits the comparison of the intervention outcomes, potentially confounding the interpretation of the intervention’s true efficacy. Third, this study will assess only the short-term and midterm effectiveness of the intervention (ie, 3 months and 6 months after treatment initiation), which precludes the evaluation of long-term smoking abstinence (eg, 12 months) and relapse rates. Finally, the study is conducted in a campus setting, where participants are primarily students who are well educated and may be more responsive to mHealth interventions, limiting the generalizability of the findings to other populations.

## Abbreviations

CONSORT: Consolidated Standards of Reporting Trials
IM: Instant messaging
mHealth: Mobile health

## Data Availability

Data of this study will be available from the corresponding author upon reasonable request after the main results of the study have been published.
